# cGMP and PKGI are required for vascular BMP signaling

**DOI:** 10.1186/1471-2210-11-S1-P65

**Published:** 2011-08-01

**Authors:** Raphaela Schwappacher, Thuan Diep, Gerry Boss, Renate Pilz

**Affiliations:** 1 Department of Medicine, University of California at San Diego, San Diego, CA 92093, USA

## Background

Maintenance of vascular homeostasis depends on phenotypic switching of vascular smooth muscle cells (VSMCs) during development, vascular injury repair, and disease. In healthy blood vessels, VSMCs exhibit a differentiated, ‘contractile’ phenotype, but in diseased vascular tissue or after vascular injury, they de-differentiate into a ‘synthetic’ state, characterized by decreased smooth muscle (SM)-specific gene expression and increased proliferation and motility [1].

## Results

Although still controversial, a large number of studies indicate that the NO/cGMP/PKGI pathway inhibits proliferation and de-differentiation of VSMCs. Subcultured primary VSMCs undergo de-differentiation to a ‘synthetic’ phenotype with both reduced SM-specific gene expression and loss of PKGI expression. A more ‘contractile’ phenotype can be regained by restoring PKGI [2]. PKGI stimulates SM-specific gene expression through regulation of the cystein-rich LIM only protein CRP4 that cooperates with SRF-containing transcription complexes [3]. But the mechanism how cGMP/PKGI regulates SM-specific phenotype is incompletely understood. In C2C12 myoblasts, PKGI phosphorylates the BMP type II receptor (BMPRII); in response to BMP-2, PKGI dissociates from the receptor, associates with the activated Smad1/4 complex, translocates to the nucleus, and forms a complex with Smads and the general transcription factor TFII-I to collaboratively activate transcription [4]. In the vascular system, BMP signaling inhibits proliferation and migration of VSMCs, and upregulates SM-specific genes, through Smad-dependent [5-8] and/or Smad-independent pathways [9-11]. Interestingly, heterozygous germline mutations within BMPRII can cause pulmonary arterial hypertension (PAH), a disease characterized by thickening of pulmonary arteries due to abnormal proliferation, migration, and/or apoptosis of VSMCs and endothelial cells [12]. Considering these data we hypothesize that PKGI promotes the differentiated ‘contractile’ phenotype of VSMCs at least in part through enhancing vascular BMP/Smad signaling. Indeed we found that cGMP/PKGI promoted Smad1/5 activation and BMP target gene expression in VSMCs and SM precursor cells. Pharmacological or siRNA-mediated inhibition of the NO/cGMP/PKGI pathway not only suppressed BMP-induced upregulation of SM-specific gene transcription, but also abrogated the anti-proliferative and anti-migratory effects of BMP on VSMCs. Furthermore preliminary data suggest that Smad crosstalk with other transcriptional regulators is involved.

## Conclusion

Our data imply that within the vasculature, PKGI is a critical regulator of the VSMC differentiation-promoting effects of BMP. The integration of cGMP/PKGI pathway into BMP/Smad signaling in the vascular system might provide new insight into the mechanisms of vascular remodeling in diseases such as atherosclerosis, vascular restenosis and pulmonary hypertension.
